# Study of the Effects of Several SARS-CoV-2 Structural Proteins on Antiviral Immunity

**DOI:** 10.3390/vaccines11030524

**Published:** 2023-02-23

**Authors:** Rong Yue, Fengyuan Zeng, Danjing Ma, Ziyan Meng, Xinghang Li, Zhenxiao Zhang, Haobo Zhang, Qi Li, Langxi Xu, Zhenye Niu, Dandan Li, Yun Liao, Guorun Jiang, Li Yu, Heng Zhao, Ying Zhang, Longding Liu, Qihan Li

**Affiliations:** Yunnan Key Laboratory of Vaccine Research and Development for Severe Infectious Diseases, Institute of Medical Biology, Chinese Academy of Medicine Sciences & Peking Union Medical College, Kunming 650118, China

**Keywords:** SARS-CoV-2, structural protein, innate immune, specific T-cell response, humoral immunity

## Abstract

The severe acute respiratory syndrome coronavirus 2 (SARS-CoV-2) Spike (S) protein is a critical viral antigenic protein that enables the production of neutralizing antibodies, while other structural proteins, including the membrane (M), nucleocapsid (N) and envelope (E) proteins, have unclear roles in antiviral immunity. In this study, S1, S2, M, N and E proteins were expressed in 16HBE cells to explore the characteristics of the resultant innate immune response. Furthermore, peripheral blood mononuclear cells (PBMCs) from mice immunized with two doses of inactivated SARS-CoV-2 vaccine or two doses of mRNA vaccine were isolated and stimulated by these five proteins to evaluate the corresponding specific T-cell immune response. In addition, the levels of humoral immunity induced by two-dose inactivated vaccine priming followed by mRNA vaccine boosting, two homologous inactivated vaccine doses and two homologous mRNA vaccine doses in immunized mice were compared. Our results suggested that viral structural proteins can activate the innate immune response and elicit a specific T-cell response in mice immunized with the inactivated vaccine. However, the existence of the specific T-cell response against M, N and E is seemingly insufficient to improve the level of humoral immunity.

## 1. Introduction

Severe acute respiratory syndrome coronavirus 2 (SARS-CoV-2), as a member of the coronavirus family beta-group [1], possesses a single-stranded negative-sense RNA genome associated with a nucleocapsid composed of four structural proteins and the viral membrane [2]. The global pandemic produced by this virus in 2019 led to massive deaths and the virus represents the greatest threat to global public health [3,4]. It is known that the spike (S) protein, which is a structural protein consisting of two subunits S1 and S2 [5], is a critical viral antigen that elicits the production of neutralizing antibodies, so most drugs and vaccines are designed against it. Although neutralizing antibodies against the S protein have been identified as the gold standard for the assessment of the immune response in individuals vaccinated with various vaccines, investigating the antigenic role of other viral structural proteins in antiviral immunity will be important for the development of a new generation of vaccines with an improved design that employs new SARS-CoV-2 antigens [6], especially a debate about antibody-dependent enhancement with coronavirus disease 2019 (COVID-19) vaccines and optimization of immunization strategies in the face of the increasing emergence of variants. Currently, abundant data regarding the analysis of the T-cell response against viral structural proteins, especially the nucleocapsid (N) protein, in a large population immunized with vaccines and some COVID-19 convalescence patients affirm the existence of a specific T-cell response against the N protein that is related to antiviral immunity [7,8,9,10,11]. Besides, other structural proteins, such as the membrane (M), N and envelope (E) proteins, affect not only the formation of the virus particle, but also antiviral immunity, although the mechanism is not exactly clear [12]. Nevertheless, limited reports suggest that the specific immune response against these structural proteins is probably involved in effective protection in infected patients and vaccines [13,14]. For instance, the N protein abundantly expressed and induced early humoral and cellular immune responses [15,16]. In addition, the M glycoprotein is a negative regulator of the innate immune response [17] and E protein is sensed by toll like receptor 2 (TLR2), leading to inflammatory responses [18]. One of the latest studies shows a diversified immune response recognizing distinct multi-structural-protein was induced by an inactivated SARS-CoV-2 vaccine and efficiently tolerated the mutations characterizing the Omicron lineage, comparable with the sole spike immune response induced by mRNA vaccine [19]. However, the synergistic effect of each protein on specific anti-S antibodies is not well understood. Thus, in this study, we aimed to clarify the immunological relationship of several structural proteins and host immunity through their interaction in their stimulation of the innate immune system and adaptive immune system. Our results suggested that these viral structural proteins, including M, N and E, enable activating the innate immune response, leading to elevated levels of some important immune regulators and eliciting specific T-cell response in mice immunized with inactivated vaccines. However, the existence of the specific T-cell response against M, N and E is seemingly insufficient to increase synergistically the level of antibody against the S antigen, whether upon primary immunization or boosting. This observation suggested that the immunogenic role of M, N and E proteins requires further investigation.

## 2. Materials and Methods

### 2.1. Cells, Viruses and Vaccines

16HBE cells, Vero cells and HEK293-ACE2 cells were obtained from the Institute of Medical Biology (IMB), Chinese Academy of Medical Sciences (CAMS). 16HBE cells and HEK293-ACE2 cells were cultured in Dulbecco’s modified Eagle’s medium (DMEM, Gibco, Grand Island, NY, USA) supplemented with penicillin, streptomycin and 10% fetal bovine serum (FBS, Sigma, St. Louis, MO, USA) at 37 °C with 5% CO_2_. Vero cells were cultured in minimum Eagle’s medium (MEM, Thermo Fisher Scientific, Waltham, MA, USA) supplemented with 10% newborn bovine serum (NBS, Sigma, St. Louis, MO, USA). The Wuhan-Hu-1 strain used for the neutralizing antibody assay was provided by the National Institute for Viral Disease Control and Prevention, China. The inactivated SARS-CoV-2 vaccine was developed by the IMB, CAMS. The mRNA vaccine was developed by Stemirna Therapeutics. The inactivated and mRNA vaccines used in this study were developed based upon the sequence of the Wuhan Hu-1 strain (GenBank: MN908947). The mRNA vaccine contains the whole S protein sequence.

### 2.2. Animal and Ethics

Four-week-old female Balb/c mice were purchased from Vital River (Beijing, China) and housed in a specific pathogen-free facility at the IMB, CAMS. The room temperature was maintained at approximately 25 °C during the experiments. Food and water were readily available. All animals were fully under the care of veterinarians at the IMB, CAMS. All animal experiments were performed according to the National Institutes of Health Guide for the Care and Use of Laboratory Animals, with approval from the Institutional Animal Care and Use Committee of the IMB, CAMS (approval number: DWSP 202003 005).

### 2.3. Plasmid Construction and Transfection

Based on the Wuhan-Hu-1 strain (GenBank: MN908947) and a reverse-transcribed cDNA library, the gene sequences of the S1, S2, M, N and E proteins of SARS-CoV-2 were synthesized by polymerase chain reaction (PCR, PrimeSTAR^®^ Max DNA Polymerase, TaKaRa Bio, Tokyo, Japan). Plasmid pcDNA3.1 (+) was digested with Kpn1 (QuickCut™ Kpn1, TaKaRa Bio, Tokyo, Japan). Primers were designed, and then obtain the inserted fragments with His-tag behind the target fragment by PCR. The inserted fragments were made recombinant with the linearized vector (ClonExpress Ultra One Step Cloning Kit, Vazyme, Nanjing, China). Detailed information regarding primers for constructing plasmids are listed in Supplementary Tables S1 and S2. 16HBE cells were subcultured into six-well plates with approximately 1 × 10^5^ cells per well and cultured overnight at 37 °C with 5% CO_2_ until the 16HBE cells grew to 60–80%. Five plasmids encoding the Wuhan-Hu-1 strain SARS-CoV-2 S1, S2, M, N and E proteins were transfected into 16HBE cells with transfection reagent (jetPRIME, Polyplus-transfection, Illkirch-Graffenstaden, France).

### 2.4. Western Blots

Forty-eight hours after transfection, the cells were lysed, and the total cell proteins were separated by 4–20% PAGE (SurePage, GenScript, Naning, China) and transferred to the PVDF membranes. The membranes were blocked with 5% skim milk (BD, Sparks, MD, USA). Then, the membranes were treated with mouse anti-His tag monoclonal antibody (Bioss antibodies, Beijing, China) and horseradish peroxidase (HRP)-conjugated goat-anti-mouse antibody (Beyotime, Shanghai, China). Finally, the PVDF membranes were developed with ECL hypersensitive chemiluminescence reagent (Beyotime, Shanghai, China) and placed in a Bio-Rad gel imager for exposure.

### 2.5. Cytokine Analysis (q-RT-PCR)

Five plasmids encoding the Wuhan-Hu-1 strain SARS-CoV-2 S1, S2, M, N and E proteins were transfected into 16HBE cells with transfection reagent (jetPRIME, Poly-plus-transfection, Illkirch-Graffenstaden, France), and the supernatant was discarded at 10, 12, 15, 20 and 24 h. The cells were lysed with 1 mL of TRIzol Universal Reagent (Tiangen, Beijing, China). Total RNA was extracted according to the instructions. RNA was amplified using a One Step TB Green^®^ PrimeScript™ PLUS RT—PCR Kit (TaKaRa Bio, Tokyo, Japan). The specific primers used are listed in Supplementary Table S3.

### 2.6. Animal Vaccination Protocol

**Figure 1:**
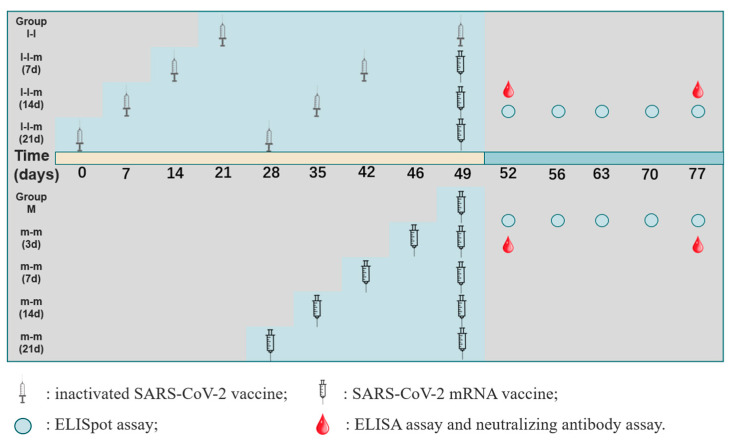
Schematic depicting the vaccination protocol. Group I-I (n = 15) was vaccinated with two doses of inactivated SARS-CoV-2 vaccine (28-day interval), and groups I-I-m (7 d), I-I-m (14 d) and I-I-m (21 d) (n = 15 per group) were, respectively, vaccinated with the third dose of mRNA booster on days 7, 14 and 21 after the second dose. Group m (n = 10) was vaccinated with a dose of mRNA vaccine, and groups m-m (3 d), m-m (7 d), m-m (14 d) and m-m (21 d) (n = 10 per group) were vaccinated with the second dose of mRNA booster on days 3, 7, 14 and 21. Control mice were injected with phosphate-buffered saline (PBS) alone. Spleens of immunized animals were collected on the 3rd, 7th, 14th, 21st and 28th days after the last immunization for an ELISpot assay. Serum samples were collected on the 3rd and 28th days for ELISA and neutralizing antibody assays (n = 2–3 per group).

The animal experiments have been divided into two parts. In the first part, female Balb/c mice (n = 60) were randomly divided into four groups. The first dose of inactivated vaccine (30 U, 100 μL) was intradermally administered, and the second dose of inactivated vaccine (28 days apart, group I-I, n = 15) was intradermally immunized, followed by intramuscularly immunization of mRNA vaccine (10 μg, 100 μL) on days 7, 14 and 21 (group I-I-m (7 d), group I-I-m (14 d) and group I-I-m (21 d), n = 15 per group). In the second part, female Balb/c mice (n = 50) were randomly divided into five groups, the first dose of mRNA vaccine (10 μg, 100 μL, group m, n = 10) was intramuscularly administered, and the second dose of mRNA vaccine was intramuscularly administered 3, 7, 14 and 21 days after the first dose (group m-m (3 d), group m-m (7 d), group m-m (14 d) and group m-m (21 d), n = 10 per group). Two or three mice in each group were euthanized at each time point for blood and spleen samples on the 3rd, 7th, 14th, 21st and 28th days after the last immunization (Figure 1).

### 2.7. ELISpot Assay

The spleens were isolated under sterile conditions, and the splenic lymphocytes suspension were prepared according to the instructions of the lymphocyte separation solution (Dakewe Biotech, Beijing, China). Splenic lymphocytes were assessed with an ELISpot kit for IFN-γ (or IL-4, IL-17) (MABTECH, Cincinnati, OH, USA). A total of 5 × 10^5^ splenic lymphocytes were inoculated in each well of IFN-γ and IL-4 plates, and IL-17 plates were seeded with 1 × 10^6^ splenic lymphocytes per well. Then, 1 μg of stimulant (five proteins: SARS-CoV-2 S1 (Sanyou, Shanghai, China, NCBI reference sequence: YP_009724390.1), S2 (Sino Biological, Beijing, China, NCBI reference sequence: YP_009724390.1), M (AtaGenix, Wuhan, China, NCBI reference sequence: YP_009724393.1), N (Sanyou, Shanghai, China, NCBI reference sequence: YP_009724397.2) and E (Sino Biological, Beijing, China, NCBI reference sequence: YP_009724392.1)) was added to each well. After incubation at 37 °C for 36 h in a 5% CO_2_ incubator, the cells and culture medium were discarded, and the plates were developed. Spot counts were performed using an automated ELISpot reader (CTL, Shaker Heights, OH, USA).

### 2.8. ELISA

The S1, S2, M, N and E proteins (same with the protein in Section 2.7) of the SARS-CoV-2 Wuhan-Hu-1 strain were utilized to coat 96-well ELISA plates (Corning, NY, USA) at a concentration of 0.1 μg per well and incubated overnight at 4 °C. Diluted serum and standard samples were added to 96-well plates precoated with specific antibody. After washing, horseradish peroxidase-conjugated antibody (Thermo Fisher, Waltham, MA, USA) and 3,3′,5,5′-tetramethylbenzidine reagent (Solarbio, Beijing, China) were added successively for signal development. The stop solution was then added (Solarbio, Beijing, China), and the absorbance at 450 nm was read with a microplate reader. The specific IgG titers were determined by end titration utilizing the reciprocal of the lowest serum dilution that produced an OD value 2.1-fold greater than that in the prebleed. In addition, commercialized anti-SARS-CoV-2 (Omicron B.1.1.529) antibody IgG titer serologic assay kit was used to test the S1-specific IgG titers (Acrobiosystems, Newark, DE, USA) according to the manufacturer’s protocol.

### 2.9. Neutralizing Antibody Assay

We purchased pseudoviruses based on the original Omicron strain (B1.1.529) (Acrobiosystems, Newark, DE, USA). To determine the neutralizing activity of serum samples, heat-inactivated serum samples were subjected to 20-fold serial dilutions starting from 1:20 to 1:5120 with a 25 μL dilution of pseudovirus added to each well according to the manufacturer’s protocol. Then, 100 μL of a HEK293T-ACE2 cell suspension (5 × 10^5^ cells/mL) was added and incubated for 48 h at 37 °C in a 5% CO_2_ atmosphere. Next, the fluorescence detection reagent was prepared (PerkinElmer, Waltham, MA, USA) and incubated according to the instructions, and the fluorescence was measured by a microplate reader (BIOTEK, Winooski, VT, USA). Then, we selected several groups to carry out a genuine virus-neutralizing antibody assay against the original Wuhan-Hu-1 strain virus. The inactivated serum was serially diluted two-fold from 1:4 to 1:128 and the genuine virus were mixed with a titer of 100 CCID_50_ (50% cell culture infectious dose) and incubated at 37 °C for 2 h. The samples were then inoculated into Vero cells and incubated for 7 days at 37 °C in a 5% CO_2_ incubator for cytopathic effect assessment. The end-point neutralization titers were defined through 50% plaque reduction assays.

### 2.10. Statistical Analysis

All the data are expressed as the mean and standard deviation (SD). Significant differences between groups were analyzed by Scheirer–Ray–Hare test (SPSS 25.0) or one-way ANOVA (and nonparametric or mixed, GraphPad Prism 9.0.0), and *p* < 0.05 was considered to indicate statistical significance. Graphs were plotted using GraphPad Prism 9.0.0.

## 3. Results

### 3.1. Innate Immune Response Elicited by Various SARS-CoV-2 Structural Proteins in Epithelial Cells

**Figure 2:**
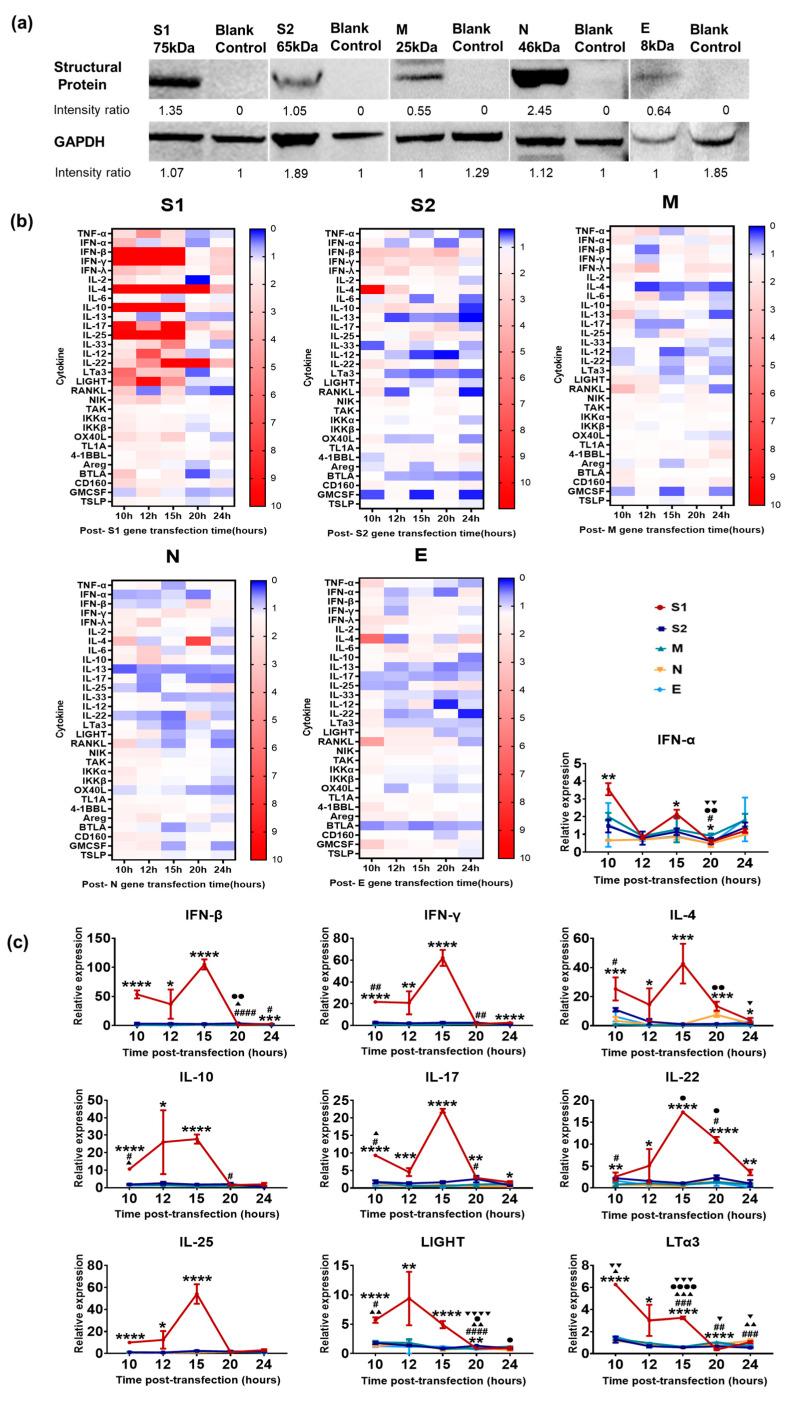
Innate immune response elicited by various SARS-CoV-2 structural proteins in 16HBE cells. (**a**) Western blot analysis to examine the expression of recombinant plasmids. 16HBE cells were transiently transfected with five recombinant plasmids and cell lysate was harvested 48 h later. (**b**,**c**) Expression of innate/inflammatory cytokines in 16HBE cells after transfection at different times. The relative expression levels of innate/inflammatory cytokines in 16HBE cells were normalized to their levels in the blank control group (transfection blank pcDNA3.1(+)) using the comparative Ct (ΔΔCt) method. Scheirer–Ray–Hare test was conducted. The data are shown as the mean ± SD based on data from two independent experiments. *, S1 vs. control. #, S2 vs. control. ▲, M vs. control. ●, N vs. control. ▼, E vs. control. * *p* < 0.05, ** *p* < 0.01, *** *p* < 0.001, **** *p* < 0.0001, ^#^ *p* < 0.05, ^##^ *p* < 0.01, ^###^ *p* < 0.001, ^####^ *p* < 0.0001, ^▲^ *p* < 0.05, ^▲▲^ *p* < 0.01, ^▲▲▲^ *p* < 0.001, ^●^ *p* < 0.05, ^●●^ *p* < 0.01, ^●●●●^ *p* < 0.0001, ^▼^ *p* < 0.05, ^▼▼^ *p* < 0.01, ^▼▼▼^ *p* < 0.001, ^▼▼▼▼^ *p* < 0.0001.

Previous data indicated that SARS-CoV-2 infection initiated in epithelial cells of the respiratory tract leads to the activation of the cellular innate immune system and associated local inflammatory reactions [20,21,22], and then promotes the activation of specific adaptive immunity [23]. This inference further logically suggests that viral structural proteins are recognized as pathogen-associated molecular patterns by cellular pattern recognition receptors and elicit alterations in the transcriptional profile of some important immune signaling molecules [24]. Based upon this understanding, we cloned the genes encoding viral S1, S2, M, N and E and then constructed expression vectors using a pcDNA3.1(+) plasmid, which were expressed in 16HBE cells (Figure 2a). Furthermore, 16HBE cells, originating from bronchial epithelial tissue, were transfected with these five expression plasmids and were collected at 10, 12, 15, 20 and 24 h post-transfection for transcriptional profile analysis. Approximately 30 signaling molecules related to innate/inflammatory reactions were detected using q-RT-PCR. The results showed that the S1 protein elicited very strong expression of some molecules, including members of the interferon family and cytokines (Figure 2b); among them, the expression of IFN-α, IFN-β, IFN-γ, IL-4 and IL-10 was upregulated by dozens or hundreds of times compared with that in the control, while the expression of LTα3 and LIGHT, which are related to antigen presenting cell activation, was obviously upregulated (Figure 2c). Conversely, S2, M, N and E protein expression in 16HBE cells elicited lower transcription of these 30 molecules (Figure 2b,c). These results suggested that several SARS-CoV-2 structural proteins, except S1, produced limited stimulation of the cellular innate immune response.

### 3.2. Structural Proteins Elicit a Specific T-Cell Response in Mice Immunized with Inactivated SARS-CoV-2 Vaccine

**Figure 3:**
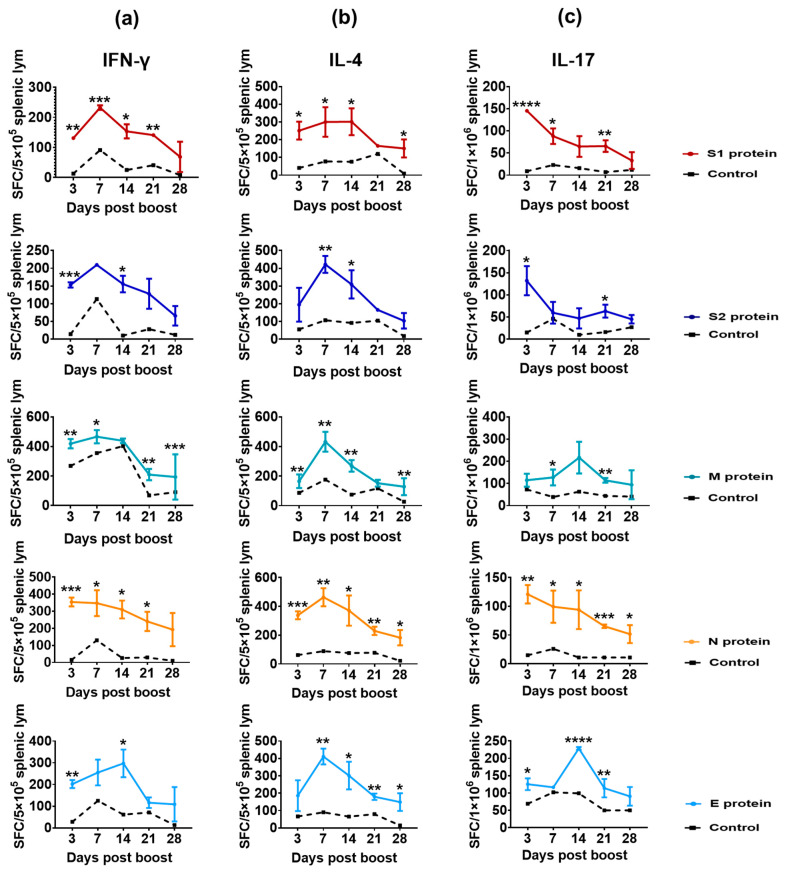
Specific T-cell responses in mice immunized with inactivated SARS-CoV-2 vaccine (n = 15), analyzed by ELISpot assay. (**a**–**c**) The ELISpot responses show IFN-γ-, IL-4- and IL-17-secreting T cells among splenic lymphocytes after stimulation with the antigenic proteins S1, S2, M, N and E. The black dotted line represents the control group. Scheirer–Ray–Hare test was conducted. Bars represent the mean ± SD. * *p* < 0.05, ** *p* < 0.01, *** *p* < 0.001, **** *p* < 0.0001 vs. control.

In many previous studies on the immunological index in vaccines or patients with COVID-19, the critical indicator was the level of neutralizing antibodies against the S protein, which was defined as an indicator of immune efficacy and immune protection [25,26]. However, the data about immune persistence in vaccines and convalescent patients also suggested that viral structural proteins, especially N protein, might be involved in immune protective efficacy and/or assist in maintaining immunity persistence [7,8,9,10,11]. Here, Balb/c mice, compared with C57BL/6 mice, which could produce a stronger humoral response for easier to observe and compare [27], immunized twice with an inactivated SARS-CoV-2 vaccine, were assayed for specific T-cell responses against various structural proteins at days 3, 7, 14, 21 and 28 post-boost immunization using antigenic proteins S1, S2, M, N and E. The results indicated that these five proteins induced a specific T-cell response (Figure 3), in which the specific T-cell response of IFN-γ peaked at days 3–14 post-boost (Figure 3a), and those with specificity for IL-4 peaked at day 7 (Figure 3b), while the specific antibodies against these viral structural proteins were elicited (Supplemental Figure S1). These results suggested that five structural proteins presented antigenic stimulation to the immune system in mice immunized with the inactivated vaccine. The result for the specific IL-17 T-cell response against the five proteins elicited in mice also supported this conclusion (Figure 3c).

### 3.3. Immunization with mRNA Vaccine for the S Gene Induces a Specific T-Cell Response against Only the S1 and S2 Proteins

**Figure 4:**
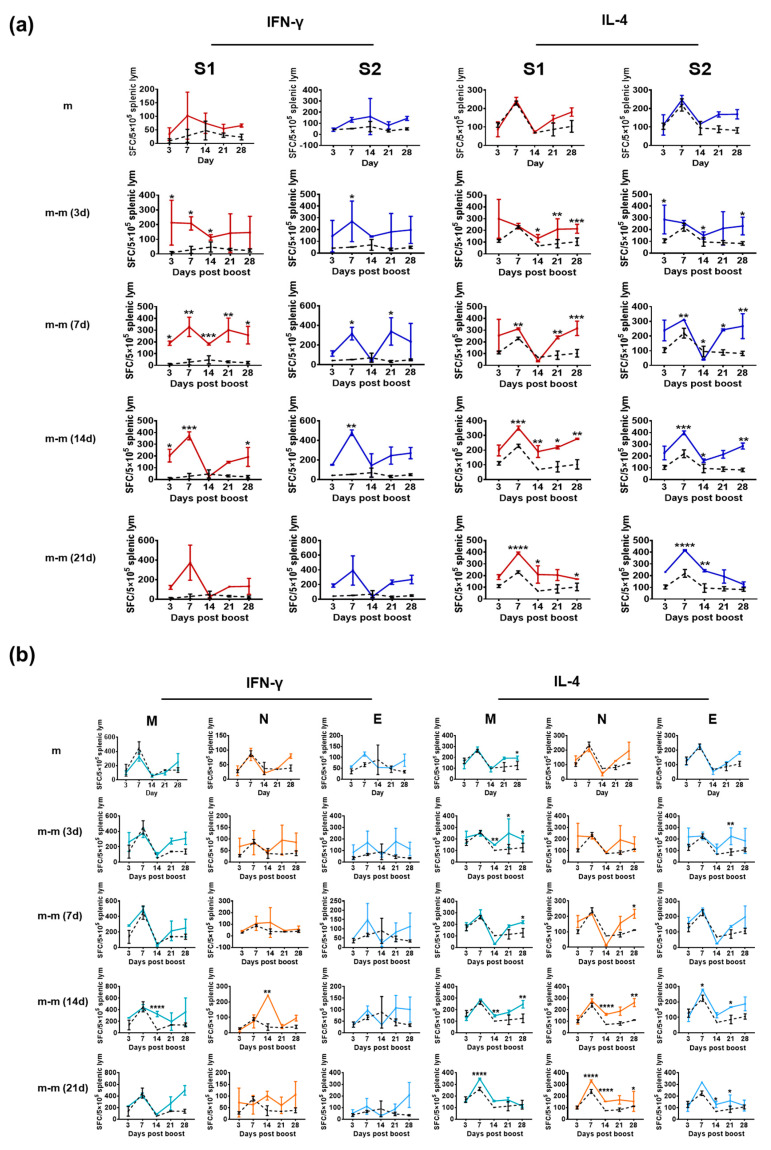
Specific T-cell responses in mice immunized with SARS-CoV-2 mRNA vaccine analyzed by ELISpot assay. Groups m-m (3 d), m-m (7 d), m-m (14 d) and m-m (21 d) (n = 10 per group) received booster immunization on days 3, 7, 14 and 21 after the first mRNA vaccine dose, respectively. The black dotted line represents the control group. (**a**) The results of the ELISpot assay specific for IFN-γ and IL-4 suggested specific T-cell responses against S1 and S2. (**b**) The results of the ELISpot assay specific for IFN-γ and IL-4 suggested specific T-cell responses against M, N and E. Scheirer–Ray–Hare test was conducted. Bars represent the mean ± SD. * *p* < 0.05, ** *p* < 0.01, *** *p* < 0.001, **** *p* < 0.0001 vs. control.

Since the licensing of administration of the SARS-CoV-2 mRNA vaccine in 2020, the obvious advantage of this vaccine type in stimulating a high neutralizing antibody response has promoted its large application worldwide [28]. Immunological surveillance of the vaccinated population has suggested that specific T-cell responses for IFN-γ and IL-4 against the S protein are elicited and associated with variations in antibody levels against the S antigen [29,30], which was inferred to indicate that the specific T-cell responses against viral antigen proteins are involved in antiviral immunity, especially immune protection [31,32]. Based on these data, we investigated the specific T-cell responses against various proteins and their relationship in mice immunized with mRNA vaccine (containing the whole S protein sequence). The results of an ELISpot assay specific for IFN-γ and IL-4 suggested that certain specific T-cell responses against S1 and S2 increased the post-vaccine boost and varied based on different intervals (different groups) between the primary and boost immunizations (Figure 4a). As expected, no specific T-cell responses against M, N and E were detected (Figure 4b) and no antibody responses were found (Supplemental Figure S2). These results suggest that the specific T-cell response elicited by the S antigen does not produce cross reactivity to the other three structural proteins, and no specific antibodies against these proteins were found.

### 3.4. Antibody Response Elicited by Two Doses of mRNA Vaccine or Two Doses of Inactivated Vaccine Followed by an mRNA Vaccine Boost in Mice

**Figure 5:**
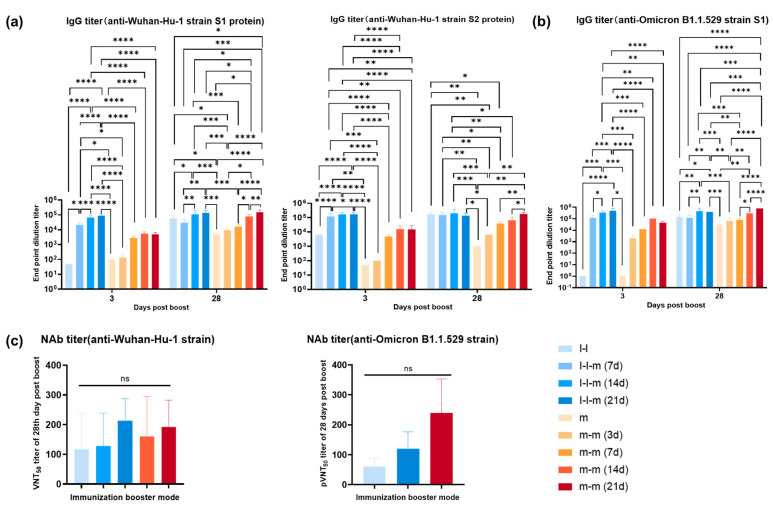
Antibody responses elicited by two doses of an mRNA vaccine or two doses of an inactivated vaccine followed by an mRNA vaccine boost. (**a**) The specific IgG antibody levels against Wuhan-Hu-1 strain S1 and S2 were detected by ELISA at 3 days and 28 days post-boost immunization. Scheirer–Ray–Hare test was conducted. (**b**) The specific IgG antibody levels against Omicron strain (B.1.1.529) S1 were detected by ELISA at 3 days and 28 days post-boost immunization. Scheirer–Ray–Hare test was conducted. (**c**) Pseudovirus-neutralizing antibody assay against the Omicron strain (B.1.1.529) and virus-neutralizing antibody assay against the Wuhan-Hu-1 strain. One-way ANOVA (nonparametric or mixed) was conducted. Bars represent the mean ± SD. Group I-I (n = 15) was vaccinated with two doses of inactivated SARS-CoV-2 vaccine (28-day interval), and groups I-I-m (7 d), I-I-m (14 d) and I-I-m (21 d) (n = 15 per group) were, respectively, vaccinated with the third dose of mRNA booster on days 7, 14 and 21 after the second dose. Group m (n = 10) was vaccinated with a dose of mRNA vaccine, and groups m-m (3 d), m-m (7 d), m-m (14 d) and m-m (21 d) (n = 10 per group) were vaccinated with the second dose of mRNA booster on days 3, 7, 14 and 21. Bars represent the mean ± SD. * *p* < 0.05, ** *p* < 0.01, *** *p* < 0.001, **** *p* < 0.0001. ns, no significance.

In an immunological study of SARS-CoV-2 vaccines with certain S neutralizing antigens, immune effects have been defined quantified based on the levels of neutralizing antibodies against the S antigen in serum [33]. In this case, the evaluation of the specific T-cell response against structural antigens, except the S antigen, depends on the relationship between the anti-S antibody titer and these specific T-cell responses [34], especially how these specific T-cell responses are involved in clinical protection against the pandemic caused by variants of concern in vaccinated individuals or patients. Therefore, our current work concerns the association of specific T-cell responses against M, N and E with the antibody responses against the S antigen. Our work, with different immunization schedules, suggested that based on distinct anti-S antibody levels elicited by the inactivated vaccine and mRNA vaccine (Figure 5a), the mRNA vaccine boost for immunized mice initially vaccinated with both vaccines led to an obvious rise in the level of antibodies against the S antigen, with similar titers (Figure 5a), and the different boost schedules resulted in distinct antibody levels (Figure 5a). However, even with the substantial increase in anti-S antibody levels at days 14 and 21 post-mRNA vaccine boost in immunized mice showing positive specific T-cell responses against M, N and E after two immunizations with the inactivated vaccine, the antibody titer did not higher than that elicited in mice immunized with two doses of the mRNA vaccine, which showed a specific T-cell response against only the S protein (Figure 5a). Although ELISA detection specific for the Omicron B.1.1.529 strain showed that boosting with the mRNA vaccine after immunization with two doses of the inactivated vaccine led to a titer of 1:400,000 in immunized mice, the two mRNA vaccine immunizations elicited higher titers (Figure 5b). Further detection using a pseudovirus-neutralizing antibody assay against the Omicron B.1.1.529 strain and a virus-neutralizing antibody assay against the Wuhan-Hu-1 strain also confirmed this difference (Figure 5c). This difference seems to suggest that the existence of specific T-cell responses against M, N and E may not improve the elicitation of an antibody response against S.

## 4. Discussion

Based on the constant change of COVID-19 pandemic, more attention has focused on the comprehensive analysis of immune response induced by various viral proteins, including structural proteins [35]. Immunological studies analyzing the SARS-CoV-2 infectious process and vaccine immune response have not yet elucidated whether the specific T-cell responses against structural proteins, including the S protein, coordinate and/or enhance the humoral immunity targeting the S protein or whether that effect is a critical effective component of immune protection against SARS-CoV-2 infection [36]. Generally, specific T-cell responses against viral structural proteins lead to immune capability to limit viral spread in tissues and enhance the production of specific antibodies [37]. The immunological observation of COVID-19 patients and individuals immunized with an inactivated vaccine has also indicated ubiquitous specific T-cell responses, especially against the N protein [13,38]. Here, our work based on eukaryotic recombination vectors expressing all structural proteins in respiratory epithelial cells showed that, among all structural proteins, the S1 protein, containing the receptor-binding domain, a major neutralizing antibody epitope, enables a much stronger upregulation of the transcriptional profile of innate immune signaling molecules than other viral structural proteins. The S2, M, N and E proteins presented weaker transcriptional activation of them, which implied a critical role of S1 in stimulating innate immunity. Interestingly, even the animal study using a SARS-CoV-2 inactivated vaccine with all structural proteins of S, M, N and E possessing primary immune stimulating effect, no matter the kind of vaccine to boost, indicated that these proteins enable the induction of a specific T-cell response, including responses of at least three CD4+ T cell subsets—Th1, Th2 and Th17. This hints that these structural proteins could cause a diversified immune response of host, which might clarify systemic immunity and defense of the host against SARS-CoV-2. In addition, although the anti-S antibody level in primary-by-inactivated vaccine groups boosted by the mRNA vaccine (only S antigen) still was not higher than that elicited by two doses of the mRNA vaccine, the strength of the specific T-cell response against the S antigen induced by the inactivated vaccine was similar to that induced by the two doses of the mRNA vaccine. These results suggested that either the specific T-cell response against S or those against M, N and E seemed unhelpful in enhancing the humoral immune response characterized by specific anti-S antibodies. Generally, specific antibody and T cell responses against viral proteins were thought being the indicators of immune response of host [39], and both indicators were interrelated and coordinated during antiviral immunity [40]. In accumulated data of SARS-CoV-2 studies, a clear conclusion has presented that S antigen is the dominant immunogen, which enables activating an innate and adaptive immune reaction, which is responsible for host immune against the virus infection [36]. Here, our work further suggested that although the structural proteins of the virus, except S, were capable of eliciting the identified specific T cell and antibodies response against them, they were unable to augment the anti-S neutralizing antibody. These data are supportive of the inference that the strategy the virus uses to elude immune response during its infection via possessing encoded structural protein with weaker immunogenicity might be due to its unique evolution process, and suggests that a new type of vaccine is needed. Certainly, whether specific T-cell responses against structural proteins of M, N, E play roles related to clinical immune protection needs further study. However, the results presented here suggest an immunological feature of SARS-CoV-2 antigens, which implies that there is no direct relationship between the anti-S antibody level and the specific T-cell responses against other structural proteins.

## Data Availability

All authors declare that the data files used in the current study can be made available upon reasonable request. Thirty signaling molecules related to innate/inflammatory reactions and antigen presenting cell activation were detected using q-RT-PCR.

## Supplementary Materials

The following supporting information can be downloaded at https://www.mdpi.com/article/10.3390/vaccines11030524/s1, Table S1: Primers used for obtain the gene sequences of the structural proteins, Table S2: Primers used for homologous recombination, Table S3: Primers used for q-RT-PCR. Figure S1: Antibody responses elicited by two doses of an inactivated SARS-CoV-2 vaccine, Figure S2: Antibody responses elicited by two doses of an mRNA vaccine.
