# Supplementary material for: Study of the Effects of Several SARS-CoV-2 Structural Proteins on Antiviral Immunity
Source: Vaccines (Basel). 2023 Feb 23;11(3):524. doi: 10.3390/vaccines11030524 (PMC10059745; doi:10.3390/vaccines11030524)
Supplement: Supplementary file 1 [file vaccines-11-00524-s001.zip › vaccines-2124520-supplementary proofed.pdf]

Table S1. Primers used for obtain the gene sequences of the structural proteins

Primer	Sequence (5'-3')
S1-F	CGGGGTACCATGTTTGTTTTTCTTGTTTTATTGCC
S1-R	ATTTGCGGCCGCTTAACGTGCCCGCC
S2-F	CGGGGTACCATGAGTGTAGCTAGTCAATCCATC
S2-R	ATTTGCGGCCGCTTATGTGTAATGTAATTTG
M-F	CGGGGTACCATGGCAGATTCCAAC
M-R	ATTTGCGGCCGCTTACTGTACAAGCAAAGC
N-F	CGGGGTACCATGTCTGATAATGGACCCC
N-R	ATTTGCGGCCGCTTAGGCCTGAGTTGAGTCA
E-F	CGGGGTACCATGTACTCATTCGTTTCGG
E-R	ATTTGCGGCCGCTTAGACCAGAAGATCAGGAAC

Table S2. Primers used for homologous recombination

Primer	Sequence (5'-3')
S1-F	TTTAAACTTAAGCTTATGTTTGTTTTTCTTGTTTTATTG
S1-R	AGTGGATCCGAGCTCTTAATGGTGATGGTGATGATGACGTGCCC GCC
S2-F	TTTAAACTTAAGCTTATGAGTGTAGCTAGTCAATCCATC
S2-R	AGTGGATCCGAGCTCTTAATGGTGATGGTGATGATGTGTGTAAT GTAATTTGACTCCTT
M-F	TTTAAACTTAAGCTTATGGCAGATTCCAACG
M-R	AGTGGATCCGAGCTCTTAATGGTGATGGTGATGATGCTGTACAA GCAAAGCAAT
N-F	TTTAAACTTAAGCTTATGTCTGATAATGGACCC
N-R	AGTGGATCCGAGCTCTTAATGGTGATGGTGATGATGGGCCTGAG TTGAGTCA
E-F	TTTAAACTTAAGCTTATGTACTCATTCGTTTCG
E-R	AGTGGATCCGAGCTCTTAATGGTGATGGTGATGATGGACCAGA

AGATCAGGAAC

Table S3. Primers used for q-RT-PCR

Primer	Sequence (5'-3')
Areg-F	GCCATTATGCTGCTGGATTGG
Areg-R	CTCACTCCCTGAAGACATCTCA
IFN-α-F	ACCCCTGCTATAACTATGACC
IFN-α-R	CTAACCACAGTGTAAAGGTGC
IFN-β-F	AACTCCACCAGCAGACAG
IFN-β-R	GAGAGCAGTTGAGGACATC
RANKL-F	GGAGGAAGCACCAAGTATT
RANKL-R	CCTCTCCAGACCGTAACT
IFN-γ-F	ATGAACGCTACACACTGCATC
IFN-γ-R	CCATCCTTTTGCCAGTTCCTC
TL1A-F	AAGCCAGACTCCATCACT
TL1A-R	TACCTACTTCGCATACAGAC
IFN-λ-F	GGACGCCTTGGAAGAGTCACT
IFN-λ-R	AGAAGCCTCAGGTCCCAATTC
TNF-α-F	GTGAGGAGGACGAACATC
TNF-α-R	TGAGCCAGAAGAGGTTGA
LIGHT-F	TCTTGCTGTTGTTCATTGC
LIGHT-R	CCTTCTTGGATGCTTCATTC
LTa3-F	GATGTCTGTCTGGCTGAG
LTa3-R	CCTGCTCTTCCTCTGTGT
GMCSF-F	TCCTGMCCTGAGTAGAGACAC
GMCSF-R	TGCTGCTTGTAGTGGCTGG
IL-2-F	TCCTGTCTTGCATTGCACTAAG
IL-2-R	CATCCTGGTGAGTTTGGGATTC
IL-6-F	ACTCACCTCTTCAGAACGAATTG

IL-6-R	CCATCTTTGGAAGGTTCAGGTTG
IL-12-F	ACCAGGTGGAGTTCAAGA
IL-12-R	GCTCATCACTCTATCAATAGTC
IL-13-F	CCTCATGGCGCTTTTGTTGAC
IL-13-R	TCTGGTTCTGGGTGATGTTGA
IL-33-F	GTGACGGTGTTGATGGTAAGAT
IL-33-R	AGCTCCACAGAGTGTTCCTTG
IL-4-F	GGTCTCAACCCCCAGCTAGT
IL-4-R	GCCGATGATCTCTCTCAAGTGAT
IL-10-F	TACGGCGCTGTCATCGATTT
IL-10-R	AAGGTTTCTCAAGGGGCTGG
IL-17-F	AGATTACTACAACCGATCCACCT
IL-17-R	GGGGACAGAGTTCATGTGGTA
IL-22-F	GCTTGACAAGTCCAACTTCCA
IL-22-R	GCTCACTCATACTGACTCCGT
GAPDH-F	GCGAGATCCCTCCAAAATCAA
GAPDH-R	GTTCACACCCATGACGAACAT
CD160-F	GCTGAGGGGTTTGTAGTGTTT
CD160-R	GTGTGACTTGGCTTATGGTGA
BTLA-F	CATCTTAGCAGGAGATCCCTTTG
BTLA-R	GACCCATTGTCATTAGGAAGCA
IL-25-F	CAGGTGGTTGCATTCTTGGC
IL-25-R	GAGCCGGTTCAAGTCTCTGT
TSLP-F	ATGTTCGCCATGAAAACTAAGGC
TSLP-R	GCGACGCCACAATCCTTGTA
OX40L-F	GGTCAGGTCTGTCAACTCCTT
OX40L-R	CATCCAGGGAGGTATTGTCAGT
IKKα-F	ATACAGCGAGCAGATGAC
IKKα-R	CAACCTCAGCATAGTGGAT

IKKβ-F	CATTGTTGTTAGCGAAGACT
IKKβ-R	GCCAGGACACTGTTAAGAT
4-1BBL-F	ACTCCTGGACTTAGACGAT
4-1BBL-R	GCTGGCACATTACAGATG
NIK-F	CCGAGAAGAAGTCCACTG
NIK-R	GTCTGCTTGTCCTCCATC
TAK-F	CCAACCAGAGTCGCAATC
TAK-R	GCAAGTCACATAGCAGAAGA

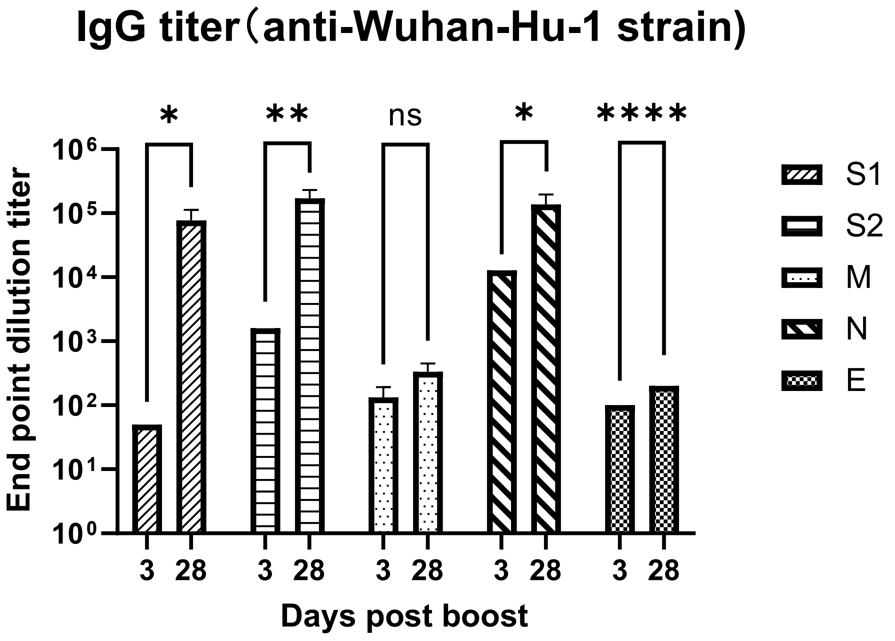

Figure S1. Antibody responses elicited by two doses of an inactivated SARS-CoV-2 vaccine. The specific IgG antibody levels against Wuhan-Hu-1 strain S1, S2, M, N and E were detected by ELISA at 3 days and 28 days post-boost immunization. Scheirer-Ray-Hare test was conducted. Bars represent the mean ± SD (n=3). *P < 0.05, **P < 0.01, ****P < 0.0001. ns, no significance.

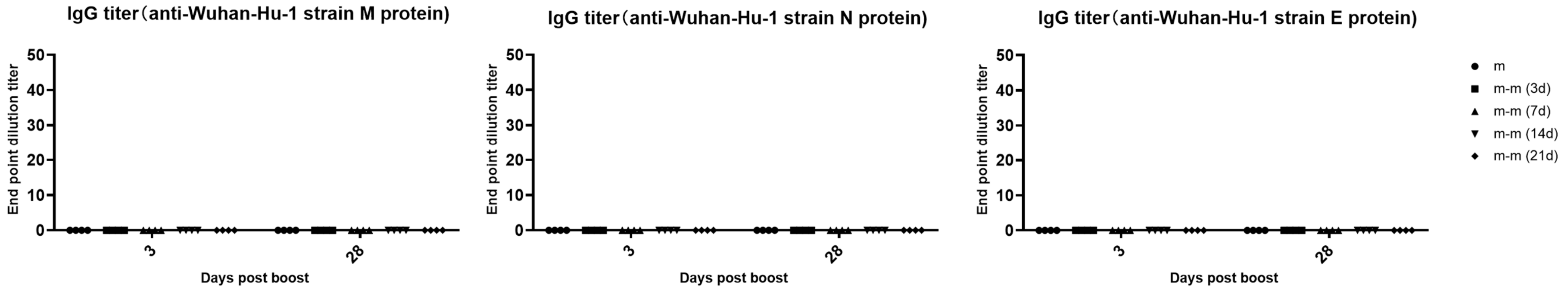

Figure S2. Antibody responses elicited by two doses of an mRNA vaccine. The specific IgG antibody levels against Wuhan-Hu-1 strain M, N and E were detected by ELISA at 3 days and 28 days post-boost immunization.
