# Supplementary figures and images for: Study of the Effects of Several SARS-CoV-2 Structural Proteins on Antiviral Immunity
Source: Vaccines (Basel). 2023 Feb 23;11(3):524. doi: 10.3390/vaccines11030524 (PMC10059745; doi:10.3390/vaccines11030524)

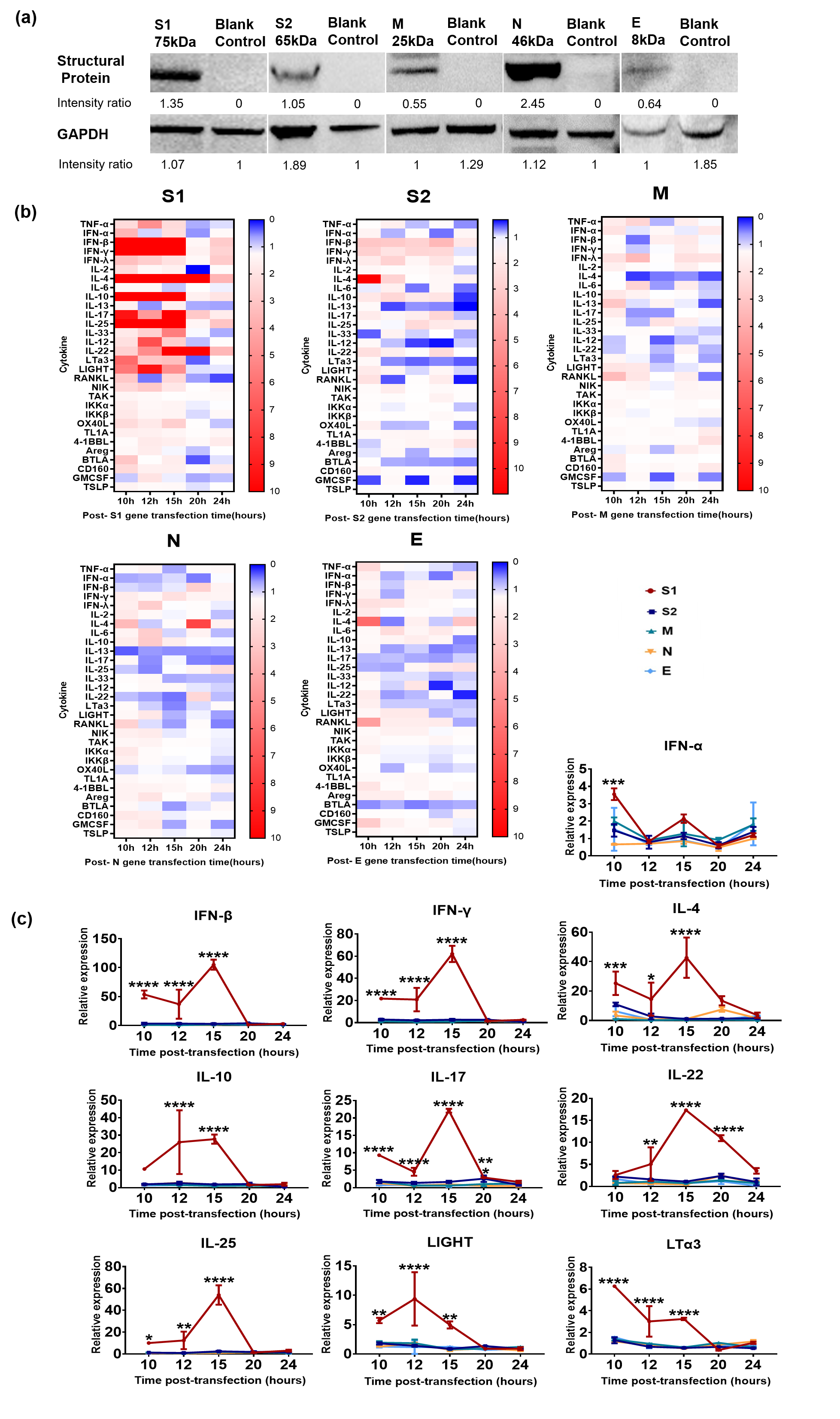

Supplement: Supplementary file 1 [file vaccines-11-00524-s001.zip › WB figures/intensity ratio-2.tif]

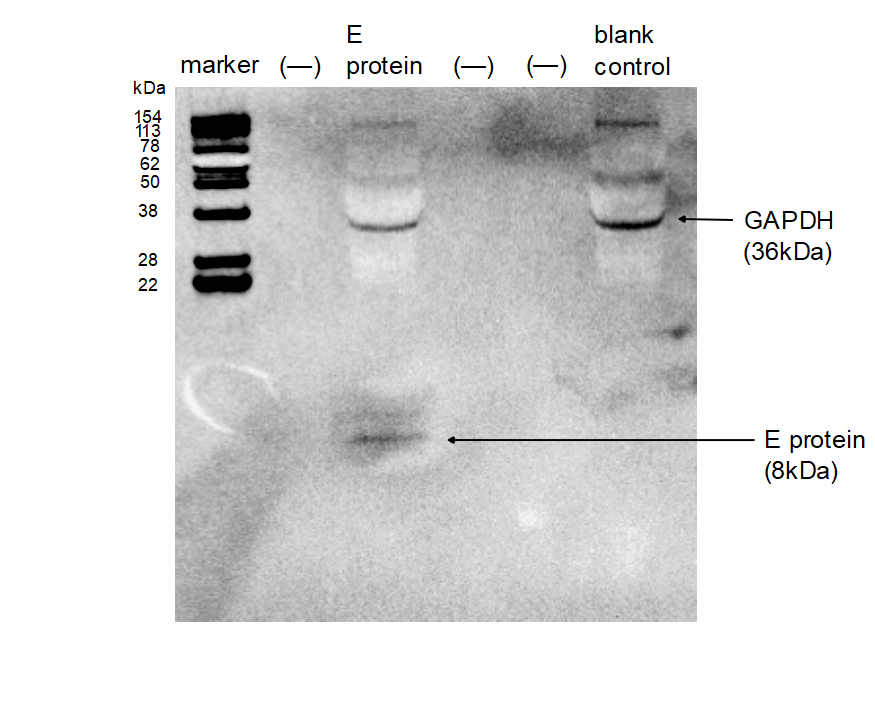

Supplement: Supplementary file 1 [file vaccines-11-00524-s001.zip › WB figures/whole blot-E protein.tif]

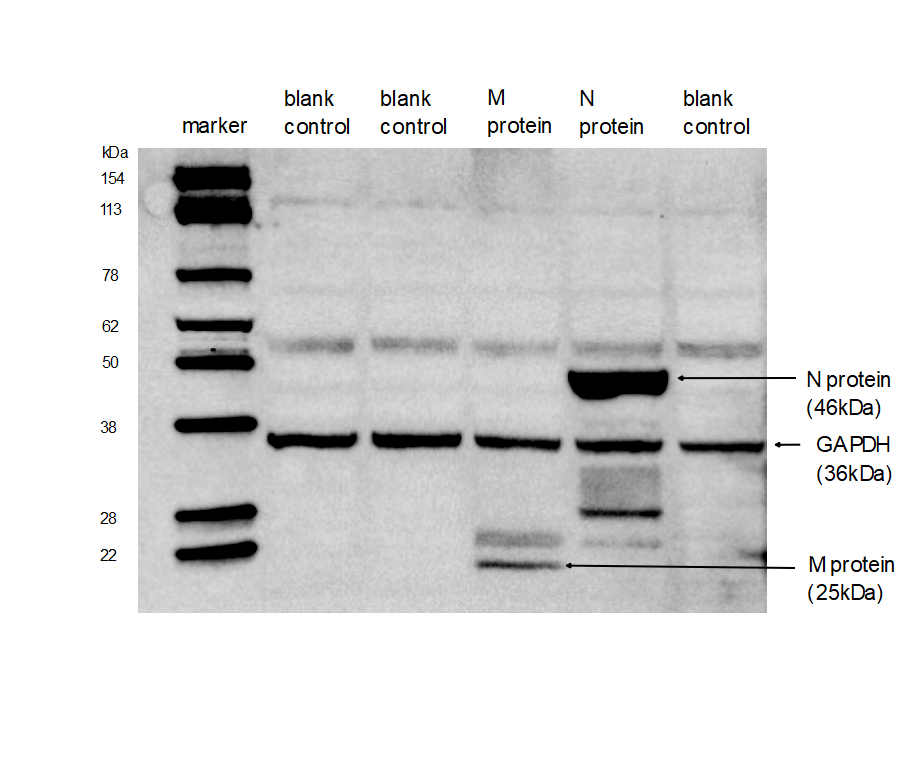

Supplement: Supplementary file 1 [file vaccines-11-00524-s001.zip › WB figures/whole blot-M protein and N protein.tif]

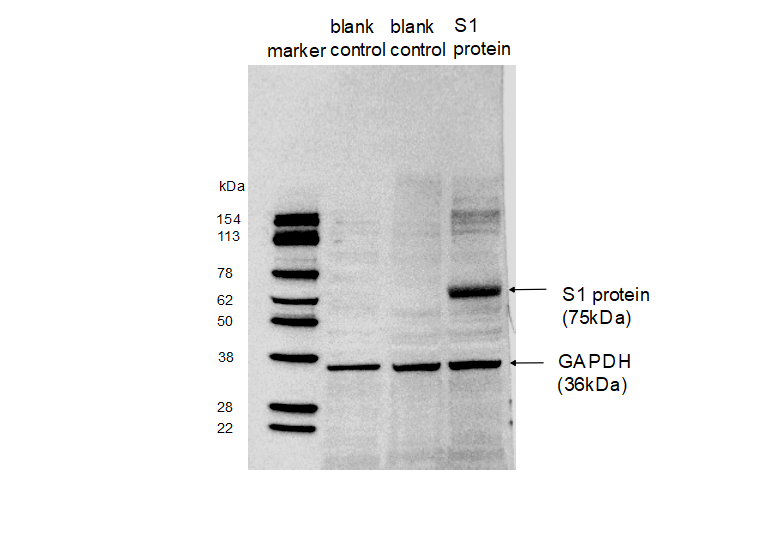

Supplement: Supplementary file 1 [file vaccines-11-00524-s001.zip › WB figures/whole blot-S1 protein.tif]

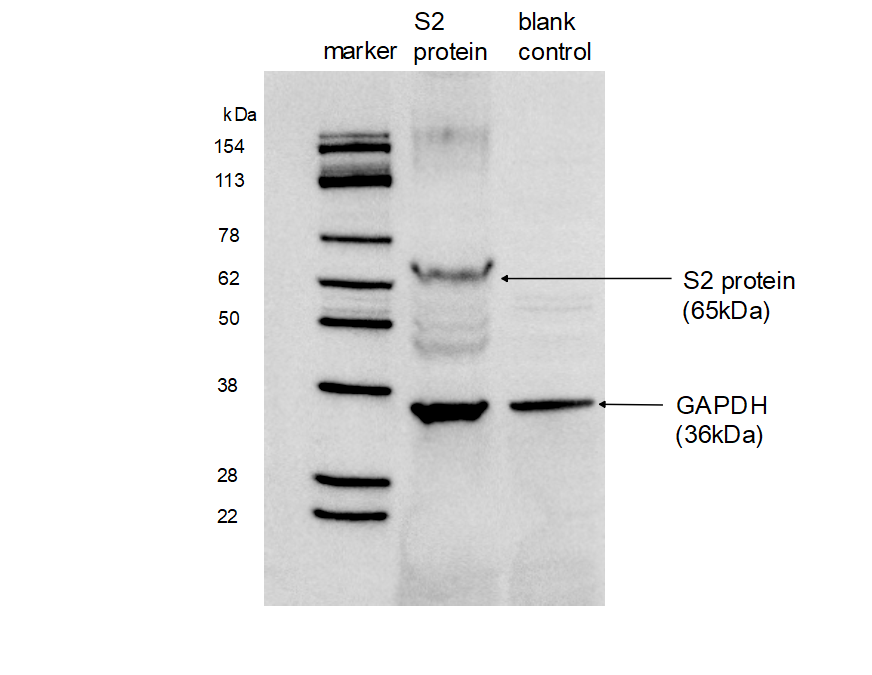

Supplement: Supplementary file 1 [file vaccines-11-00524-s001.zip › WB figures/whole blot-S2 protein.tif]
